# Molecular surveillance of nasopharyngeal carriage of *Streptococcus pneumoniae* in children vaccinated with conjugated polysaccharide pneumococcal vaccines

**DOI:** 10.1038/srep23809

**Published:** 2016-04-05

**Authors:** Anne L. Wyllie, Alienke J. Wijmenga-Monsuur, Marlies A. van Houten, Astrid A. T. M. Bosch, James A. Groot, Jody van Engelsdorp Gastelaars, Jacob P. Bruin, Debby Bogaert, Nynke Y. Rots, Elisabeth A. M. Sanders, Krzysztof Trzciński

**Affiliations:** 1 Paediatric Immunology and Infectious Diseases, Wilhelmina Children’s Hospital, University Medical Center Utrecht, Utrecht, the Netherlands; 2 Centre for Infectious Disease Control, National Institute for Public Health and the Environment (RIVM), Bilthoven, the Netherlands; 3 Spaarne Gasthuis Academie, Spaarne Gasthuis, Hoofddorp, the Netherlands; 4 Regional Laboratory of Public Health, Haarlem, the Netherlands

## Abstract

Following the introduction of pneumococcal conjugate vaccines (PCVs) for infants, surveillance studies on *Streptococcus pneumoniae* carriage have proven valuable for monitoring vaccine effects. Here, we compared molecular versus conventional diagnostic methods in prospective cross-sectional surveillances in vaccinated infants in the Netherlands. Nasopharyngeal samples (n = 1169) from 11- and 24-month-old children, collected during autumn/winter 2010/2011 and 2012/2013, were tested by conventional culture for *S. pneumoniae.* DNA extracted from all culture-plate growth was tested by qPCR for pneumococcal-specific genes (*lytA*/*piaB*) and selected serotypes (including PCV13-serotypes). qPCR significantly increased the number of carriers detected compared to culture (69% vs. 57%, *p* < 0.0001). qPCR assays targeting vaccine-serotypes 4 and 5 proved non-specific (results excluded). For serotypes reliably targeted by qPCR, the number of serotype-carriage events detected by qPCR (n = 709) was 1.68× higher compared to culture (n = 422). There was a strong correlation (rho = 0.980; *p* < 0.0001) between the number of serotypes detected using qPCR and by culture. This study demonstrates the high potential of molecular methods in pneumococcal surveillances, particularly for enhanced serotype detection. We found no evidence of a hidden circulation of vaccine-targeted serotypes, despite vaccine-serotypes still significantly contributing to invasive pneumococcal disease in unvaccinated individuals, supporting the presence of a substantial *S. pneumoniae* reservoir outside vaccinated children.

*Streptococcus pneumoniae* is a commensal of the upper respiratory tract and the causative agent of respiratory infections and life-threatening invasive pneumococcal disease (IPD)^1^. The main virulence factor of *S. pneumoniae* is its polysaccharide capsule; non-encapsulated strains are virtually absent among strains causing IPD^2^. With over 90 capsular types (serotypes) identified based on capsule chemistry and immunogenicity^3^, pneumococci represent one of the most antigenically diverse species of respiratory bacterial pathogens, although only a small subset of serotypes actually dominate in carriage or disease at any given time and location^2^,^4^,^5^,^6^.

Since the year 2000, pneumococcal conjugate vaccines (PCVs) have been introduced worldwide to combat the burden of disease in young children, by targeting the serotypes most common in disease^7^. Initially targeting seven serotypes (4, 6B, 9V, 14, 18C, 19F, 23F; Prevenar^®^, PCV7), vaccine coverage has been expanded to cover ten (PCV7-serotypes and serotypes 1, 5, 7F; Synflorix^®^, PCV10) and thirteen (PCV10-serotypes and serotypes 3, 6A, 19A; Prevenar-13^®^, PCV13) serotypes. PCV-implementation not only lowered incidence and mortality of disease caused by vaccine-serotypes (VT) in immunised children, but as a result of herd protection VT disease also diminished among unvaccinated individuals in the population^8^,^9^ demonstrating that infant vaccination reduces VT circulation in the whole population. It also indicates that young children are a major reservoir and a key source of transmission of pneumococci^1^,^10^. However, in infants, VT carriage has been almost completely replaced by carriage of non-vaccine-serotypes (NVT) with similar trends observed in the unvaccinated population, the phenomenon described as serotype replacement. As a result, the decline in VT disease has been followed by an increase in disease caused by NVTs^1^,^5^,^11^,^12^,^13^,^14^.

In the Netherlands, PCV7 was introduced into the National Immunisation Programme (NIP) for all infants born after March 2006^15^. PCV10 replaced PCV7 for all children born after February 2011^16^. The effects of PCV on pneumococcal carriage in vaccinated infants were investigated in carriage surveillance studies conducted before PCV-introduction and 3, 4.5 and 6.5 years following^15^,^16^,^17^,^18^. Three years after the introduction of PCV7, VT carriage had virtually disappeared in both children and parents, while strains of the additional PCV10 serotypes 1, 5 and 7F remained low in number^17^. However, accompanying the decrease of VTs an increase in carriage of NVTs was observed, with serotype 19A most prevalent in PCV7-vaccinated children closely followed by serotypes 6C, 15B/C and 11A^18^.

The current gold standard diagnostic method for the detection of *S. pneumoniae* in carriage surveillances is conventional culture of a deep trans-nasal nasopharyngeal swab followed by serotyping of any cultured strain by the Quellung method^19^. This method is labour-intensive, time-consuming and not suited for the detection of co-carriage of multiple pneumococcal strains, when present^20^,^21^. Recently developed molecular diagnostic methods address at least some of the limitations of conventional culture, however validation of these methods and studies investigating their reliability in field surveillances remain limited^22^. In particular, the specificity of serotype detection in polymicrobial samples has been questioned following reports of confounding organisms generating false positive signals^23^,^24^,^25^,^26^.

Here, we had a unique opportunity to compare the detection of *S. pneumoniae* carriage and serotype distribution, using both conventional culture and the sensitive molecular method of qPCR^22^, applied to the same nasopharyngeal samples collected from healthy children aged 11- or 24-months in two large cross-sectional studies conducted in the Netherlands as part of continuous nationwide surveillance on effects of PCV. Our results demonstrate the power of the molecular method for enhanced detection of both overall pneumococcal carriage and of circulating serotypes and underline important considerations for using these methods in surveillance on serotype carriage.

## Methods

### Study design

Two cross-sectional studies were performed among 11- and 24-month-old children during the autumn/winter seasons of 2010/2011 and 2012/2013, 4.5 and 6.5 years following the implementation of PCV in the Dutch NIP in a 3 + 1 schedule (2, 3, 4 and 11 months of age). With 1320 children enrolled in total, both studies included 330 healthy infants per age group. Samples from 11-month-old children were collected before the fourth vaccine dose. In 2010/2011, 11-month-olds were vaccinated with PCV7 and in 2012/2013 with PCV10. All infants sampled at 24-months of age were fully vaccinated with PCV7. Detailed descriptions of the study populations and carriage results from conventional culture-based surveillance studies are available elsewhere^16^,^18^.

Both studies were reviewed by acknowledged Dutch National Ethics Committees. The study conducted in 2010/2011 was judged as an observational study without invasive measurements and needed no approval^18^. The study conducted in 2012/2013 contained invasive measurements and was approved by METC Noord-Holland (NL40288.094.12)^16^. Written informed consent was obtained from all parents and both studies were conducted in accordance with the European Statements for Good Clinical Practice and the declaration of Helsinki of the World Health Medical Association.

### Sample collection

Trans-nasal nasopharyngeal samples were obtained according to standard procedures^19^ using a flexible, sterile swab^18^. Samples were collected by trained study personnel during scheduled home visits. Swabs were immediately placed in 1 ml Amies transport medium^15^ and transferred within 8 hours to the Regional Laboratory of Public Health in Haarlem.

### Detection of pneumococcal carriage using the conventional culture method

On arrival, samples were processed for pneumococcal detection by the standard, conventional culture diagnostic approach^15^,^19^. Briefly, one colony of pneumococcus-like morphology per plate was sub-cultured (more if distinct morphotypes were observed) and processed using conventional methods of species determination (optochin susceptibility and bile solubility assays)^16^,^18^. *S. pneumoniae* strains cultured were serotyped using the Quellung method^15^. Following, all remaining bacterial growth on the primary culture plate (blood agar supplemented with gentamicin 5 mg/l) was harvested and stored frozen as previously described^23^,^27^. These samples were considered to be culture-enriched for *S. pneumoniae*. In 2010/2011, all culture plates were transferred in weekly intervals for harvest at the University Medical Center Utrecht (UMCU) research laboratory^27^. In 2012/2013 cultures were harvested on a daily basis at the diagnostic laboratory in Haarlem and stored frozen at −80 °C before being transported on dry ice for further processing at UMCU.

### Detection of pneumococcal carriage using qPCR

As previously described, DNA was extracted from 200 μl of all culture-enriched samples^23^ and tested in quantitative-PCR (qPCR) assays targeting two pneumococcal genes *lytA*^28^ and *piaB*^27^. We have previously referred to the latter assay as targeting *piaA*^23^,^25^,^27^. Due to changes in annotation since the sequence was first published (GenBank accession number AF338658.1), the gene is now recognised as *piaB*. Samples were classified as positive for *S. pneumoniae* when C_*T*_ values for both targeted genes were ≤35^27^.

### Re-culturing of samples with discordant culture and molecular detection results

Samples classified culture-negative yet positive for *S. pneumoniae* by molecular methods, were re-cultured in a second attempt to isolate pneumococcus as previously described^27^.

### Determination of sample serotype composition using qPCR

All DNA templates were tested for the presence of serotype-specific sequences, using a panel of primers and probes, targeting 18 pneumococcal serotypes/serogroups, including those targeted by PCV13 and selected others: 1, 3, 6A/B/C/D, 7A/F, 9A/N/V, 10A/B, 14, 15A/B/C, 19A, 22A/F, 23F, 33A/F/37^29^, 11A/D, 16F^30^, 4, 5, 18B/C and 19F^29^,^30^. Samples were considered positive for presence of the targeted sequence when the serotype/serogroup-specific signal was ≤35 C_*T*_^30^.

### Statistics

Statistical analyses were conducted using GraphPad Prism v6.02 for Windows (GraphPad Software, La Jolla, CA, USA). Unless otherwise stated, statistical significance was determined using Fisher’s Exact test and defined as *p* < 0.05.

## Results

Of the 1320 infants enrolled in the study, 1169 (89%) nasopharyngeal samples were analysed. These samples were from 291 11-month-olds and 293 24-month-old children in 2010/2011 and 292 11-month-olds and 293 24-month-olds in 2012/2013.

### Pneumococcal carriage rates

Results of *S. pneumoniae* detection by conventional culture and molecular methods are reported in Table 1. Altogether, 676 *S. pneumoniae* strains were isolated from 670 of 1169 (57%) children at the initial culture step, whereas 801 of 1169 (69%) infants were positive for *S. pneumoniae* after DNA extracted from culture-enriched samples was tested with *lytA/piaB* qPCRs, with the number of carriage events detected significantly higher for qPCR-based results (Chi-Square, *p* < 0.0001). Overall, 803 of 1169 (69%) infants were identified as carriers of *S. pneumoniae* either by conventional culture or by the qPCR-based method. This included 668 (83%) carriers identified with both methods, 133 (17%) detected only with molecular method and two (0.2%) infants positive for pneumococci only by culture. Samples from these two infants were positive for strains non-typeable by the Quellung method, yet only positive for *lytA* (not *piaB*) in qPCR analysis, thus by our study criteria, classified as negative for pneumococci by the molecular method^27^.

Following the analysis of individual study sub-groups, the significant increase in the number of carriers detected with the molecular versus culture method was observed only in infants sampled in 2010/2011. The fractions of infants identified as colonised with *S. pneumoniae* based on the culture method in the subsets analysed in this study and original study groups were not significantly different (see Supplementary Table 1)^16^,^18^.

### Reculturing of samples discordant for *S. pneumoniae* presence by molecular and culture methods

To confirm the presence of pneumococci in samples that were initially culture-negative but positive by the subsequent molecular testing, culture-enriched samples from all 66 qPCR-positive, culture-negative 11–month-old children (approximately half of all 133 children detected as carriers only by the molecular method) were re-cultured and carefully inspected for pneumococcal presence as previously described^27^. Under re-examination, live *S. pneumoniae* were isolated from 52 (79%) of these 66 samples, pointing at under-detection by conventional culture as the main source of discordance between the results of conventional culture and molecular methods.

### Serotype carriage

*S. pneumoniae* strains representing 38 serotypes (plus 11 non-typeable strains) were cultured from the samples analysed in this study. Detailed results are listed in Supplementary Table 2. Our panel of eighteen serotype/serogroup-specific qPCR assays targeted 25 (66%) of these 38 serotypes, with some qPCR assays unable to distinguish between individual serotypes within the target serogroup. DNA extracted from all 1169 culture-enriched samples, both positive and negative for pneumococcal presence, was tested with these qPCR assays.

As we previously reported for samples collected from children and aged adults^23^,^25^, here we also observed false positive signals in samples negative for pneumococcal presence when using the qPCR assays published by Azzari *et al.*^29^, targeting VTs 4, 5, 18B/(C) and 19F. Therefore, these four qPCRs were considered unreliable and results were excluded from analysis. When applying the corresponding assays as published by Pimenta *et al.*^30^, assays targeting serotypes 4 and 5 also showed a lack of specificity. However, the assays designed to detect serogroup 18B/C-specific and serotype 19F-specific sequences generated no false positive signals^30^. Thus, sixteen assays (targeting thirty serotypes) were considered to be specific when used to test samples in this study.

Results for individual serotype detection by both the conventional and molecular methods are presented in Table 2. Detailed results per study group are reported in Supplementary Table 3. The number of individuals qPCR-positive for a serotype/serogroup was higher or equal compared to the number of serotype carriers detected using culture method alone in all assays except for that targeting serogroup 22A/F^29^. This assay was therefore classified as lacking in sensitivity according to our study criteria and results were excluded from further analysis.

**Figure 1:**
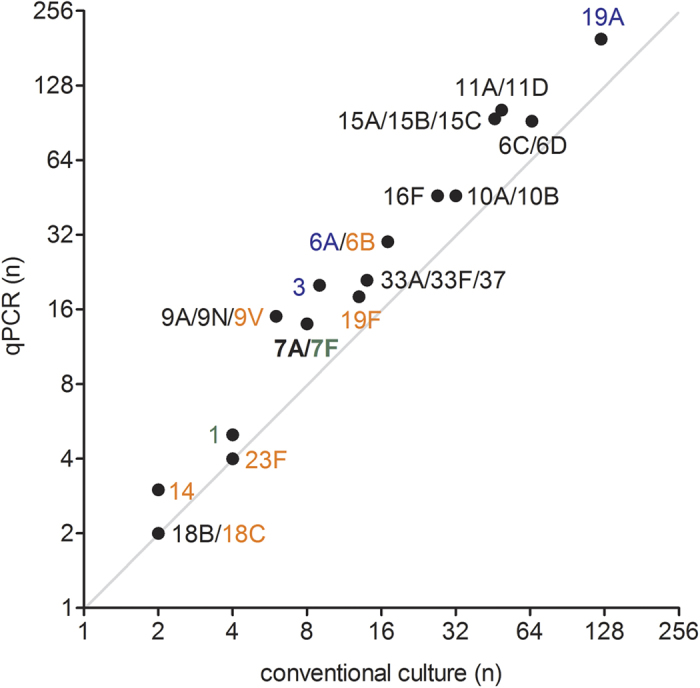
Pneumococcal serotypes carried by all infants included in the study, as detected by conventional culture compared to detection by the molecular method of qPCR. The graph depicts the correlation between the number of strains cultured and the overall number of samples positive for the corresponding serotype when tested by the molecular method, for the subset of serotypes targeted by reliable qPCR assays (Spearman’s rho = 0.980; *p* < 0.0001). Font colour indicates serotypes targeted by the pneumococcal conjugate vaccines: seven-valent (PCV7, orange), ten-valent (PCV10, green) and thirteen-valent (PCV13, blue) or non-vaccine serotypes (black).

For the remaining fifteen qPCR assays, targeting 28 serotypes in total (21 of which were detected by culture, see legend to Table 3 for serotypes), the number of serotypes detected in carriage by qPCR strongly correlated with the prevalence according to the culture results (Spearman’s rho = 0.980; *p* < 0.0001) (Fig. 1). For this subset of serotypes, the increase in overall number of serotype carriage events detected by any method in the study compared to culture data alone was higher (Chi-Square, *p* < 0.05) for serotypes that infants were not vaccinated against, namely serotypes 1, 3, 6C/D, 7A/F, 9A/N, 10A/B, 11A/D, 15A/B/C, 16F, 19A and 33A/F/37 (overall 1.71× increase from 384 to 657 carriage events from 1169 children sampled, Chi-Square *p* < 0.0001) as compared to the carriage of serotypes the infants were vaccinated against, namely 14, 18C, 19F and 23F (overall 1.29× increase from 21 to 27 carriage events, Chi-Square *p* = 0.38). PCV10-VTs 1 and 7F were considered in the analysis as serotypes not vaccinated against due to their absence in the only PCV10-vaccinated infants, the 11-month-olds sampled in the second study period (see Supplementary Table 3). Furthermore, since qPCR could not discriminate between VT 6B and NVT 6A, results were excluded from this comparison. Table 3 depicts the number of strains detected by conventional culture as compared to the number of serotype-specific signals detected by our panel of fifteen reliable qPCR assays. In summary, the molecular method increased the overall number of serotype-carriage events detected by a factor of 1.69 (714 serotype carriage events detected; 709 detected by qPCR versus 422 strains cultured). Co-carriage of multiple serotypes was detected in 180 (22%) of 803 infants identified as carriers of *S. pneumoniae* (Table 3). Although co-carriage rates were higher in 2010/2011 (n = 109 of 427, 26%) compared to 2012/2013 (n = 71 of 376, 19%; *p* = 0.027), the differences are difficult to interpret considering that co-carriage is likely underestimated due to the restricted coverage of serotypes targeted by molecular assays.

## Discussion

Epidemiological surveillances on pneumococcal carriage have proven highly valuable for monitoring the direct and indirect effects of vaccination, for identification of new or emerging NVTs that may pose a risk for replacement disease and for monitoring any changes to the invasiveness of circulating strains^11^,^17^,^31^,^32^. The very nature of surveillance on carriage requires methods which allow for high numbers of samples to be processed both time and cost effectively, but also with high sensitivity and specificity in order to provide accurate data^11^,^19^,^20^,^22^,^32^,^33^. Here, we applied molecular methods to detect pneumococcal carriage and serotypes present in nasopharyngeal samples of infants already tested by conventional culture which detected an average carriage rate of 57%.

We expected pneumococcal colonisation rates in infants to be underestimated when detected by the culture method alone, being less sensitive for the detection of *S. pneumoniae* present at low relative abundance in polymicrobial respiratory samples^23^,^25^,^27^. Since VTs were still contributing to IPD in the general population at the time of sample collection^13^ and since very young children are considered the main reservoir of *S. pneumoniae*^10^, we also hypothesised that testing samples with sensitive molecular methods would unveil a hidden circulation of VTs in PCV-vaccinated infants, not detected when samples were tested only by conventional culture^1^. We tested the latter hypothesis using the method recently ranked as one of the most sensitive in serotype carriage detection, namely testing DNA extracted from culture-enriched samples in a series of serotype-specific, singleplex real-time PCR assays^22^.

In line with our expectations, application of the molecular method significantly increased the overall number of pneumococcal carriers among all infants sampled in the study. However, this increase did not reach significance in the 2012/2013 study period. Nevertheless, our data clearly demonstrate the overall higher sensitivity of our molecular method for carriage detection compared to the gold standard conventional culture. Also, in line with results of our previous study in adults^27^, we were able to recover live *S. pneumoniae* from the majority of qPCR-positive samples that were classified in routine culture as negative. We attribute the failure to detect pneumococci at the primary culture step to a low relative abundance of *S. pneumoniae* among co-present streptococcal species, all morphologically similar on culture medium. As a result, colonies of *S. pneumoniae* are masked.

The improvement to overall pneumococcal detection by culture in the current study suggests that one of the applications for the molecular method could be for the initial screening of samples for positivity for *S. pneumoniae* followed by careful examination of these pneumococci-positive samples by conventional culture in order to increase the rate of recovery of live strains. In surveillance studies where carriage is suspected to be low or difficult to detect, such is in older age-groups or when oral samples are to be tested, this approach could increase carriage detection sensitivity, while saving labour time and thus increase cost effectiveness^23^,^25^,^27^.

Accompanying an increased sensitivity in overall pneumococcal carriage detection, we also expected molecular methods to improve sensitivity of serotype detection. Indeed, we observed a 1.68× higher number of serotype-specific carriage events detected by qPCR as compared to the culture-based method while testing for a subset of only 28 serotypes. In line with results from culture-based pneumococcal surveillance studies^16^, application of molecular methods also revealed that PCV10-VTs decreased in frequency among carriers between the two study periods (from n = 33, 8% of 427 to n = 13, 4% of 376, *p* = 0.01), mostly due to a decline in circulation of PCV7-VTs alone. Of the PCV10-specific VTs, only serotype 1 showed an increase (although non-significant, *p* = 0.19) in frequency among carriers. While serotype 1 is known for its cyclical appearance and disappearance from carriage in the population^5^, in 2012/2013 it was only detected in 24-month-old infants, who were not immunised with PCV10. Interestingly, we also observed a significant decline in frequency in carriage of PCV13-unique serotypes 3, 6A and 19A between 2010/2011 (n = 174, 41% of 427) and 2012/2013 (n = 92, 24% of 376, *p* = <0.0001) mainly due to the decline in serotype 19A alone (*p* = 0.0003) (Table 2). Following the initial introduction of PCV7 into infant-vaccination programmes, serotype 19A rapidly expanded in carriage^17^,^31^,^34^. Following the introduction of PCV13 however, carriage prevalence of 19A declined^35^,^36^. In the absence of PCV13 in the Dutch NIP (PCV10 replaced PCV7 in 2011), the mechanism behind the decline in 19A carriage remains obscure. We previously speculated this could be due to a naturally induced move towards equilibrium in frequency among serotypes replacing VTs in carriage six years post-PCV7 implementation in the Netherlands^16^, as described by Hanage *et al.* for Massachusetts^37^. In line with findings of culture-based pneumococcal surveillances^16^, we confirmed via molecular methods that the reduction in frequency of serotype 19A strains in carriage observed in 2012/2013 was significant only in 24-month-olds, all vaccinated with PCV7 (*p* = 0.0002), but not in 11-month-olds, vaccinated with PCV10 (*p* = 0.22) (see Supplementary Table 3). This result supports the presence of more casual relationship between PCV10-implementation and the decline in serotype 19A carriage in the Netherlands.

Detection of all serotypes simultaneously carried is of high importance for a greater understanding of PCV-induced changes to serotype circulation^22^. With the growing evidence that the carriage of secondary strains is common^23^,^34^,^38^, the contribution of unmasking to changes in serotype carriage reported post PCV-implementation is highly likely^11^,^39^. Also, it has been speculated that strains of more invasive serotypes such as 1, 7F, 12F and 14 may circulate as secondary in co-carriage, masked in presence by serotypes dominating the colonised niche^1^. However, this was not evident in the current study. Our results (Fig. 1) demonstrate that for serotypes rarely detected by conventional culture, including PCV10-specific VTs, application of the molecular method does not unveil a hidden circulation within our study population. Instead, molecular methods detected a higher circulation of those serotypes also more commonly detected by culture, indicating they are more common as both primary and secondary serotypes. Absence of a hidden circulation of VTs in vaccinated infants while VTs were still significantly contributing to IPD in unvaccinated individuals in the Netherlands, supports the presence of a substantial *S. pneumoniae* reservoir outside vaccinated children^13^. We reported evidence for this when investigating carriage of pneumococci in elderly during the winter season of 2011/2012^25^.

This study demonstrates the high potential of molecular methods in surveillance on pneumococcal carriage, particularly in regards to the greater resolution of serotype detection and importantly, the detection of multiserotype carriage^32^. However, molecular assays are mostly developed for serotyping individual pneumococcal isolates^40^,^41^,^42^ or clinical specimens positive for a single strain^43^,^44^,^45^; extensive validation of these assays is essential before they can be reliably applied to clinical samples of complex microbial profiles^22^. This may only become apparent when also testing samples negative for *S. pneumoniae.* Results from this study and our previous studies clearly demonstrate the potential for false positivity^23^,^25^ and demonstrate the need for testing all samples in serotyping assays and not just those positive for presence of pneumococci. The specificity of assays should be of particular concern when high rates of VT carriage are reported in post-PCV surveillances on *S. pneumoniae*, in which only samples pre-selected for positivity for pneumococci, are tested with molecular methods alone^46^,^47^,^48^. While recently published qPCR assays^30^ have overcome false positive signals for 18B/C and 19F, this is not the case for VTs 4 and 5. Studies continuing to utilise assays generating false positive signals could incorrectly interpret these false positive results as lack of PCV effects on carriage of VTs in the population. Furthermore, assay-sensitivity must also be considered, as demonstrated by the qPCR assay targeting serotype 22A/F. Reduced sensitivity will lead to an under-detection of circulating serotypes, particularly important if assays target VTs or NVTs increasing in prevalence following PCV-introduction. Also, we recognise that not testing for a broader range of serotypes with qPCR, including certain NVTs emerging in carriage in the vaccinated population (for example, serogroup 23 strains), as a limitation in our study. In particular, this limits the analysis of co-carriage rates, with multiserotype colonisation likely underestimated.

Through the unique settings of the current study we have further demonstrated the continued alterations to pneumococcal and serotype distribution, occurring in children aged 2 years and under, following the implementation of PCV in the Netherlands^16^,^17^,^18^. Since improved knowledge of serotypes circulating in carriage allows implementation of more accurate preventive measures against pneumococcal disease our findings are important for strategies targeting IPD. We demonstrate that while VTs now rarely circulate, their detection in carriage by conventional culture is not underestimated compared to NVTs. It suggests to us that despite host vaccination, when present, VTs are carried as primary serotypes. This is in line with results reported for animal models^49^ showing that type-specific antibodies prevent acquisition of VT strains but has little impact on already established carriage.

Our study demonstrates the need for further development and optimisation of molecular methods for pneumococcal serotype detection. For effective vaccine surveillance, molecular assays that are sensitive and specific for individual serotypes and able to detect serotypes without interference from confounding, non-pneumococcal bacteria are essential.

## Additional Information

**How to cite this article**: Wyllie, A. L. *et al.* Molecular surveillance of nasopharyngeal carriage of *Streptococcus pneumoniae* in children vaccinated with conjugated polysaccharide pneumococcal vaccines. *Sci. Rep.* **6**, 23809; doi: 10.1038/srep23809 (2016).

## Supplementary Material

Supplementary Information

## Figures and Tables

**Table 1:** Nasopharyngeal carriage of *S. pneumoniae* detected with conventional and molecular diagnostic methods in 11- and 24-month-old children.

Study year	Age (months)	n	PCV	Method of carriage detection				
				Conventional culture		Molecular method		Either method
				Number *(%)*^a^ positive	Sensitivity^b^	Number *(%)* positive	Sensitivity	Number *(%)* positive
**2010/2011**	**11**	**291**	7	150 *(52)*	0.77	194 (67)**	1.0	194 *(67)*
	**24**	**293**	7	188 *(64)*	0.81	233 (80)***	1.0	233 *(80)*
	**Combined**	**584**		338 *(58)*	0.79	427 (73)****	1.0	427 *(73)*
**2012/2013**	**11**	**292**	10	169 *(58)*	0.88	189 *(65)*	0.99	191 *(65)*
	**24**	**293**	7	163 *(56)*	0.88	185 *(63)*	1.0	185 *(63)*
	**Combined**	**585**		332 *(57)*	0.88	374 (64)*	0.99	376 *(64)*
**Overall**	****	**1169**	****	**670** ***(57)***	**0.83**	**801** (69)****	**1.0**	**803** ***(69)***

^*^p  = 0.012, ^**^p = 0.0003; ^***^p < 0.0001; ^****^Chi-Square p < 0.0001; significant difference in number of carriers detected by the molecular method as compared to conventional culture.

^a^Fraction of children identified as carriers in the study group.

^b^Fraction of all carriers identified by any method (reported in last column).

**Table 2:** Overall number of the serotype strains detected by conventional culture and serotype-specific signals detected by the molecular method (qPCR) among all 803 infants identified as carriers of *S. pneumoniae* by either method used in the study.

Serotype/serogroup	2010/2011 (n = 427)			2012/2013 (n = 376)			Overall (n = 803)		
	Culture	qPCR	Total	Culture	qPCR	Total	Culture	qPCR	Total
**1**PCV10	1	1	**1**	3	4	**4**	4	5	**5**
**3**PCV13	4	13	**13**	5	7	**7**	9	20	**20**
**4**PCV7	1^a^	NS^b^	**1**	0	NS	**0**	1	NS	**1**
**5**PCV10	1	NS	**1**	0	NS	**0**	1	NS	**1**
**6A**PCV13**/6B**PCV7	7/5^c^	22	**22**	2/3	8	**8**	9/8	30	**30**
**6C/6D**	26/0	44	**44**	39/0	48	**49**	65/0	92	**93**
**7A/7F**PCV10	0/5	11	**11**	0/3	3	**3**	0/8	14	**14**
**9A/9N/9V**PCV7	0/3/0	11	**11**	0/3/0	4	**5**	0/6/0	15	**16**
**10A/10B**	12/0	22	**22**	20/0	24	**24**	32/0	46	**46**
**11A/11D**	22/0	48	**48**	27/0	54	**54**	49/0	102	**102**
**14**PCV7	2	3	**3**	0	0	**0**	2	3	**3**
**15A/15B/15C**	2/12/14	56	**56**	4/10/4	38	**39**	6/22/18	94	**95**
**16F**	9	22	**22**	18	24	**24**	27	46	**46**
**18B/18C**PCV7	0/1	1	**1**	0/1	1	**1**	0/2	2	**2**
**19A**PCV13	76	127	**127**	48	70	**70**	124	197	**197**
**19F**PCV7	8	13	**13**	5	5	**5**	13	18	**18**
**22A/22F**	1/3	0	**4**	0/10	2	**12**	1/13	2	**16**
**23F**PCV7	4	4	**4**	0	0	**0**	4	4	**4**
**33A/33F/37**	2/3/1	10	**11**	5/2/1	11	**12**	7/5/2	21	**23**
**Other**^d^	113	–	**113**	125	–	**125**	238	–	**238**
**Total**	338	408	**528**	338	303	**442**	676	711	**970**

^PCV7^serotype targeted by all three pneumococcal conjugate vaccines (PCVs); ^*PCV10*^serotype targeted by PCV10 and PCV13 but not PCV7; ^*PCV13*^serotype targeted only by PCV13.

^a^Total number of carriers positive for the particular serotype.

^b^NS - assay considered to be non-reliable due to lack of specificity.

^c^n/n - Serotype-specific conventional culture results for serotypes indistinguishable from the serogroup when targeted by qPCR, numbers correspond to serotypes reported in the first column.

^d^Serotypes not targeted by qPCR assays available thus detected only by conventional culture.

**Table 3:** Number of pneumococcal strains detected by conventional culture and the molecular method (qPCR).

Study year	Age (m)	Number of serotype carriage events (number of different serotypes detected^a^)				Mean number of serotype-specific signals detected (Culture/qPCR)	Carriers of multiple serotypes *(% of all carriers)*
		Culture (All)	Culture (qPCR panel^b^)	qPCR (qPCR panel)	Factor increase by qPCR detection		
**2010/2011**	**11**	150 (31)	98 (17)	177 (14)	1.81	1.00/1.28	45 of 194 *(23)*
	**24**	188 (33)	121 (20)	231 (16)	1.91	1.00/1.33	64 of 233 *(27)*
	**Combined**	338 (38)	219 (21)	408 (16)	1.86	1.00/1.31	109 of 427 *(26)*
**2012/2013**	**11**	172 (26)	95 (15)	148 (12)	1.56	1.02/1.24	39 of 191 *(20)*
	**24**	166 (28)	108 (17)	153 (13)	1.42	1.02/1.19	32 of 185 (17)*
	**Combined**	338 (31)	203 (16)	301 (14)	1.48	1.02/1.22	71 of 376 (19)*
**Overall**		**676 (39)**	**422 (21)**	**709 (16)**	**1.68**	**1.01/1.27**	**180 of 803** ***(22)***

^a^Individual serotypes detected by both culture and the molecular method: 1, 3, 6A, 6B, 6C, 7F, 9N, 10A, 11A, 14, 15A, 15B, 15C, 16F, 18C, 19A, 19F, 23F, 33A, 33F and 37.

^b^Subset of serotypes tested for by qPCR: 1, 3, 6A/B/C/D, 7A/F, 9A/N/V, 10A/B, 11A/D, 14, 15A/B/C, 16F, 18B/C, 19A, 19F, 23F, 33A/F/37.

^*^p < 0.05; significant difference in the number of carriers of multiple serotypes detected in 2012/2013 as compared to 2010/2011.
