# Supplementary material for: Molecular surveillance of nasopharyngeal carriage of Streptococcus pneumoniae in children vaccinated with conjugated polysaccharide pneumococcal vaccines
Source: Sci Rep. 2016 Apr 5;6:23809. doi: 10.1038/srep23809 (PMC4820691; doi:10.1038/srep23809)
Supplement: Supplementary Information [file srep23809-s1.pdf]

Molecular surveillance of nasopharyngeal carriage of *Streptococcus pneumoniae* in children vaccinated with conjugated polysaccharide pneumococcal vaccines

Anne L. Wyllie[1], Alienke J. Wijmenga-Monsuur[2], Marlies A. van Houten[3], Astrid A.T.M. Bosch[1,3], James A. Groot[1], Jody van Engelsdorp Gastelaars[1], Jacob P. Bruin[4], Debby Bogaert[1], Nynke Y. Rots[2], Elisabeth A.M. Sanders[1,2*] and Krzysztof Trzciński[1*#]

[1]Paediatric Immunology and Infectious Diseases, Wilhelmina Children's Hospital, University Medical Center Utrecht, Utrecht, the Netherlands;

[2]Centre for Infectious Disease Control, National Institute for Public Health and the Environment (RIVM), Bilthoven, the Netherlands;

[3]Spaarne Gasthuis Academie, Spaarne Gasthuis, Hoofddorp, the Netherlands;

[4]Regional Laboratory of Public Health, Haarlem, the Netherlands.

*these authors contributed equally to this study

Supplementary Table 1. Conventional culture based detection of *Streptococcus pneumoniae* in nasopharyngeal samples collected from infants in the original studies published by Spijkerman *et al*.[1] and Bosch *et al*.[2] and in the subsets analysed in this study.

	samples positive for *S. pneumoniae*/ all samples tested in the study		
**study group**	**original study**	**analysed in this study**	***p* value**
11-month-olds/ 2010/2011[1]	173/330	150/291	0.872[a]
24-month-olds/ 2010/2011[1]	211/330	188/293	1.000[a]
11-month-olds/ 2010/2011[2]	187/330	169/292	0.808[a]
24-month-olds/ 2010/2011[2]	173/330	163/293	0.469[a]
**All**	**744/1320**	**670/1169**	**0.632[b]**

[a]Two-tailed Fisher's Exact probability test; [b]Chi-square

Supplementary Table 2. Pneumococcal serotypes detected only by the conventional method in all 803 carriers of pneumococci.

Serotypes/ Serogroups	2010/2011 11-month-olds (n=194)	2010/2011 24-month-olds (n=233)	2012/2013 11-month-olds (n=191)	2012/2013 24-month-olds (n=185)	2010/2011 Overall (n=427)	2012/2013 Overall (n=376)	Overall (n=803)
**23B**	7[a]	13	15	18	20	33	**53**
	0.036[b]	*0.056*	*0.079*	*0.097*	*0.047*	*0.088*	***0.066***
**21**	9	11	11	8	20	19	**39**
	0.046	*0.047*	*0.058*	*0.038*	*0.047*	*0.051*	***0.049***
**23A**	8	7	15	9	15	24	**39**
	0.041	*0.030*	*0.079*	*0.043*	*0.035*	*0.064*	***0.049***
**35B**	6	7	9	4	13	13	**26**
	0.031	*0.030*	*0.047*	*0.022*	*0.030*	*0.035*	***0.032***
**35F**	2	7	6	6	9	12	**21**
	0.010	*0.030*	*0.031*	*0.032*	*0.021*	*0.032*	***0.026***
**17F**	4	6	3	3	10	6	**16**
	0.021	*0.026*	*0.016*	*0.016*	*0.023*	*0.016*	***0.020***
**NT**	3	3	3	2	6	5	**11**
	0.015	*0.013*	*0.016*	*0.011*	*0.014*	*0.013*	***0.014***
**8**	4	4	0	0	8	0	**8**
	0.021	*0.017*	*0*	*0*	*0.019*	*0*	***0.010***
**31**	2	1	3	1	3	4	**7**
	0.010	*0.004*	*0.016*	*0.005*	*0.007*	*0.011*	***0.009***
**38**	2	2	0	3	4	3	**7**
	0.010	*0.009*	*0*	*0.016*	*0.009*	*0.008*	***0.009***
**24F**	0	1	3	2	1	5	**6**
	0	*0.004*	*0.016*	*0.011*	*0.002*	*0.013*	***0.007***
**29**	2	0	0	0	2	0	**2**
	0.010	*0*	*0*	*0*	*0.005*	*0*	***0.002***

**42**	0	1	1	0	1	1	**2**
	0	*0.004*	*0.005*	*0*	*0.002*	*0.003*	***0.002***
**12F**	0	1	0	0	1	0	**1**
	0	*0.004*	*0*	*0*	*0.002*	*0*	***0.001***
**Total**	**49**	**64**	**69**	**56**	**113**	**125**	**238**
	0.253	***0.275***	***0.361***	***0.303***	***0.264***	***0.332***	***0.296***

[a]total number of carriers positive for the particular serotype.

[b]fraction of carriers positive for the particular serotype among all carriers identified in a study group with any method.

Supplementary Table 3. Overall number of serotype strains detected by conventional culture and serotype-specific signals detected by molecular method (qPCR) per study group, among all 803 infants identified as carriers of *S. pneumoniae* by either method used in the study.

Serotypes/ serogroups	**2010/2011 11-month-olds (n=194)**			**2010/2011 24-month-olds (n=233)**			**2012/2013 11-month-olds (n=191)**			**2012/2013 24-month-olds (n=185)**		
	Culture	qPCR	**Total**	Culture	qPCR	**Total**	Culture	qPCR	**Total**	Culture	qPCR	**Total**
**1**[PCV10]	0[a]	0	**0**	1	1	**1**	0	0	**0**	3	4	**4**
	0[b]	*0*	***0***	*0.004*	*0.004*	***0.004***	*0*	*0*	***0***	*0.016*	*0.022*	***0.022***
**3**[PCV13]	2	6	**6**	2	7	**7**	4	5	**5**	1	2	**2**
	0.010	*0.031*	***0.031***	*0.009*	*0.030*	***0.030***	*0.021*	*0.026*	***0.026***	*0.005*	*0.011*	***0.011***
**4**[PCV7]	0	NS[c]	**0**	1	NS	**1**	0	NS	**0**	0	NS	**0**
	0	-	***0***	*0.004*	-	***0.004***	*0*	-	***0***	*0*	-	***0***
**5**[PCV10]	1	NS	**1**	0	NS	**0**	0	NS	**0**	0	NS	**0**
	0.005	-	***0.005***	*0*	-	***0***	*0*	-	***0***	*0*	-	***0***
**6A**[PCV13]**/6B**[PCV7]	3/3[d]	13	**13**	4/2	9	**9**	1/3	7	**7**	1/0	1	**1**
	0.015/0.015	*0.067*	***0.067***	*0.017/0.009*	*0.039*	***0.039***	*0.005/0.016*	*0.037*	***0.037***	*0.005/0*	*0.005*	***0.005***
**6C/6D**	13/0	19	**19**	13/0	25	**25**	18/0	24	**25**	21/0	24	**24**
	0.067/0	*0.098*	***0.098***	*0.056/0*	*0.107*	***0.107***	*0.094/0*	*0.126*	***0.131***	*0.114/0*	*0.130*	***0.130***
**7A/7F**[PCV10]	0/1	5	**5**	0/4	6	**6**	0/0	0	**0**	0/3	3	**3**
	0/0.005	*0.026*	***0.026***	*0/0.017*	*0.026*	***0.026***	*0/0*	*0*	***0***	*0/0.016*	*0.016*	***0.016***
**9A/9N/9V**[PCV7]	0/3/0	9	**9**	0	2	**2**	0/2/0	2	**2**	0/1/0	2	**3**
	0/0.015/0	*0.046*	***0.046***	*0*	*0.009*	***0.009***	*0/0.011/0*	*0.011*	***0.011***	*0/0.005/0*	*0.011*	***0.016***

**10A/10B**	3	7	**7**	9	15	**15**	11	11	**11**	9	13	**13**
	0.015/0	*0.036*	***0.036***	*0.039/0*	*0.064*	***0.064***	*0.055/0*	*0.055*	***0.055***	*0.049/0*	*0.068*	***0.068***
**11A/11D**	9/0	16	**16**	13/0	32	**32**	8/0	21	**21**	19	33	**33**
	0.046/0	*0.082*	***0.082***	*0.056/0*	*0.137*	***0.137***	*0.042/0*	*0.110*	***0.110***	*0.103/0*	*0.178*	***0.178***
**14**[PCV7]	0	1	**1**	2	2	**2**	0	0	**0**	0	0	**0**
	0	*0.005*	***0.005***	*0.009*	*0.009*	***0.009***	*0*	*0*	***0***	*0*	*0*	***0***
**15A/15B/15C**	0/6/6	26	**26**	2/6/8	30	**30**	0/2/1	17	**17**	4/8/3	21	**22**
	0/0.031/0.031	*0.134*	***0.134***	*0.009/0.026/0.034*	*0.129*	***0.129***	*0/0.010/0.005*	*0.089*	***0.089***	*0.022/0.043/0.016*	*0.114*	***0.119***
**16F**	7	14	**14**	2	8	**8**	12	15	**15**	6	9	**9**
	0.036	*0.072*	***0.072***	*0.009*	*0.034*	***0.034***	*0.060*	*0.075*	***0.075***	*0.032*	*0.047*	***0.047***
**18B/18C**[PCV7]	0/0	0	**0**	0/1	1	**1**	0/1	1	**1**	0/0	0	**0**
	0	*0*	***0***	*0/0.004*	*0.004*	***0.004***	*0/0.005*	*0.005*	***0.005***	*0/0*	*0*	***0***
**19A**[PCV13]	33	48	**48**	43	79	**79**	25	37	**37**	23	33	**33**
	0.170	*0.247*	***0.247***	*0.184*	*0.339*	***0.339***	*0.131*	*0.194*	***0.194***	*0.124*	*0.178*	***0.178***
**19F**[PCV7]	4	6	**6**	4	7	**7**	3	3	**3**	2	2	**2**
	0.021	*0.030*	***0.030***	*0.017*	*0.030*	***0.030***	*0.016*	*0.016*	***0.016***	*0.011*	*0.011*	***0.011***
**22A/22F**	1/1	0	**2**	0/2	0	**2**	0/8	2	**8**	0/2	0	**2**
	0.005/0.005	-	***0.010***	*0/0.009*	-	***0.009***	*0/0.040*	-	***0.040***	*0/0.011*	-	***0.011***
**23F**[PCV7]	2	2	**2**	2	2	**2**	0	0	**0**	0	0	**0**
	0.010	*0.010*	***0.010***	*0.008*	*0.008*	***0.008***	*0*	*0*	***0***	*0*	*0*	***0***

**33A/33F/37**	1/1/1	5	**5**	1/2/0	5	**6**	3/1/0	5	**6**	1/2/1	6	**6**
	0.005/0.005/0	*0.026*	***0.026***	*0.004/0.009/0*	*0.021*	***0.026***	*0.016/0.005/0*	*0.026*	***0.031***	*0.005/0.011/0.005*	*0.032*	***0.032***
**Other**[d]	49	-	**49**	64	-	**64**	69	-	**69**	56	-	**56**
	0.252	-	***0.252***	*0.274*	-	***0.274***	*0.361*	-	***0.361***	*0.303*	-	***0.303***
**Total**	150	177	**229**	188	231	**299**	172	150	**227**	166	153	**213**

[PCV7]serotype targeted by all three pneumococcal conjugate vaccines (PCVs); [PCV10]serotype targeted by PCV10 and PCV13 but not PCV7; [PCV13]serotype targeted only by PCV13.

[a]total number of carriers positive for the particular serotype.

[b]fraction of carriers positive for the particular serotype among all carriers identified in a study group with any method.

[c]NS - assay considered to be non-reliable due to lack of specificity.

[d]n/n - Serotype-specific conventional culture results for serotypes indistinguishable from the serogroup when targeted by qPCR, numbers correspond to serotypes reported in the first column.

[d]Serotypes not targeted by qPCR assays available thus detected only by conventional culture.

REFERENCES

1. Spijkerman, J. *et al.* Long-term effects of pneumococcal conjugate vaccine on nasopharyngeal carriage of S. pneumoniae, S. aureus, H. influenzae and M. catarrhalis. *PLoS One* **7,** e39730 (2012).
2. Bosch, A. A. T. M. *et al.* Nasopharyngeal carriage of Streptococcus pneumoniae and other bacteria in the 7th year after implementation of the pneumococcal conjugate vaccine in the Netherlands. *Vaccine* **34,** 531–9 (2016).
